# An ensemble strategy that significantly improves *de novo* assembly of microbial genomes from metagenomic next-generation sequencing data

**DOI:** 10.1093/nar/gkv002

**Published:** 2015-01-13

**Authors:** Xutao Deng, Samia N. Naccache, Terry Ng, Scot Federman, Linlin Li, Charles Y. Chiu, Eric L. Delwart

**Affiliations:** 1 Blood Systems Research Institute, San Francisco, CA 94118, USA; 2 Department of Laboratory Medicine, University of California at San Francisco, San Francisco, CA 94107, USA; 3 UCSF-Abbott Viral Diagnostics and Discovery Center, San Francisco, CA 94107, USA; 4 Department of Medicine, Division of Infectious Diseases, UCSF, San Francisco, CA 94143, USA

## Abstract

Next-generation sequencing (NGS) approaches rapidly produce millions to billions of short reads, which allow pathogen detection and discovery in human clinical, animal and environmental samples. A major limitation of sequence homology-based identification for highly divergent microorganisms is the short length of reads generated by most highly parallel sequencing technologies. Short reads require a high level of sequence similarities to annotated genes to confidently predict gene function or homology. Such recognition of highly divergent homologues can be improved by reference-free (*de novo*) assembly of short overlapping sequence reads into larger contigs. We describe an ensemble strategy that integrates the sequential use of various de Bruijn graph and overlap-layout-consensus assemblers with a novel partitioned sub-assembly approach. We also proposed new quality metrics that are suitable for evaluating metagenome *de novo* assembly. We demonstrate that this new ensemble strategy tested using *in silico* spike-in, clinical and environmental NGS datasets achieved significantly better contigs than current approaches.

## INTRODUCTION

With rapidly declining cost, next-generation sequencing (NGS) approaches have become common for comprehensive pathogen identification in clinical and environmental samples. This powerful technology has numerous applications in diagnosis of infectious diseases, environmental surveillance, metagenomic analysis of human and animal microbiomes, and novel pathogen discovery (1–5). Using bioinformatics, microbial sequences are identified by comparing millions of NGS reads to sequences in publicly available reference databases. One crucial step in the analysis is *de novo* metagenome assembly of short overlapping reads into longer contigs. Successful assembly can generate long contigs or even complete genomes, which has two major advantages: (i) enhance the sensitivity to detect novel pathogens with only weak sequence homology to known pathogens by generation of long contigs (6); and (ii) reduce the cost and labor needed to manually extend new microbial genomes with polymerase chain recation.

Many assemblers have been recently categorized in a review by Miller *et* *al*. (7). One school of assemblers such as AMOS (8), CAP3 (9), Celera (10), VCAKE (11) and Newbler (12) use traditional olconsensus (OLC) algorithms which identify overlaps between various long reads and subsequently merge the read fragments into longer sequences. This approach requires pairwise evaluation of a large number of reads, which is computationally intensive. Another group of assemblers, such as SOAPdenovo2 (13), ABySS (14), Velvet (15), MetaVelvet (16) and ALLPATHS-LG (17) speed up assembly by using de Bruijn graph (DBG) algorithms. DBG methods leverage graph theory by using strings of a particular length (*k*-mer) to generate a sequence graph where each node is a (*k*-1)-mer and each edge is a *k*-mer which connects suffix and prefix nodes. For example, a 4-mer edge ATTG in a DBG connects prefix node ATT and suffix node TTG. To generate a DBG, each read is mapped as a path of *k*-mers, one base at a time. Sequence redundancy is naturally handled by the graph without affecting the number of nodes. Much greater speed over OLC assemblers is thus achieved by the DBG algorithm by avoiding pairwise comparison of all reads, which can be highly redundant. Assemblers have also been developed for specific genomic applications, such as Trinity (18), a DBG-based *de novo* transcriptome assembler, Masurca (19) a hybrid assembler that combines the concept of DBG and OLC methods and IDBA-UD, an assembler designed for uneven metagenomic applications (20). In addition to DBG and OLC approaches, other assembly algorithms have been reported, including MIRA4 (21) and Omega (22).

Many *de novo* assemblers were comprehensively evaluated by the Assemblathon1 (23), Assemblathon2 (24) and GAGE (Genome Assembly Assessment Project) (25) projects. The key lessons learned from these projects are that there is a lack of consensus from assemblies generated by different assemblers, and that there are no universal winners across different datasets. In many cases, the assemblers are able to generate good-sized contigs up to hundreds of kb. However, the tested datasets focused on NGS assembly of large human, animal or bacterial genomes from pure samples and cultures and at a high depth of coverage, whereas metagenomic samples present greater challenges because they contain a complex mixture of sequence fragments from multiple viruses, bacteria and animal/human host DNA. Based on published data from our group (1) and others (26), DBG assemblers rarely generate contigs more than a few kb in real-world metagenomic samples. Given the lack of a ‘best’ assembler, the Assemblathon papers advised against relying solely on a single assembler for any given dataset of interest. One recent paper presents a tool for comparing the performance of assemblers by scoring each assembly based on the consistency between assemblies and input reads and read pairs (27). However, it is still not clear how to optimally integrate results from multiple assemblers and derive better contigs based on a combined approach.

In this study, our goal was to evaluate current assemblers and to create a *de novo* assembly strategy tailored for analysis of metagenomic samples. In our previous report (1), we found empirically that a sequential DBG and OLC method that also incorporates partitioning was more efficient at contig assembly of viral genomes from metagenomic NGS data. Here, we formally extend these findings by rigorous comparison of common DBG and OLC assemblers and show that a two-step ensemble assembly strategy generates contigs of much higher quality than those achieved from single assemblers alone. The ensemble strategy is thus directly applicable for assembly of small viral, bacterial and eukaryotic mitochondrial genomes from a wide variety of NGS metagenomic datasets as well as from pure cultures.

## MATERIALS AND METHODS

### Datasets

Three groups of datasets named ‘*in silico*-virus spiked’, ‘pooled virus standard’ and ‘human/animal pathogens’, were used to evaluate the ensemble strategy. All datasets contained at least one target pathogen with a fully sequenced reference genome, which was used as the standard to evaluate contig size and degree of misassembly.

The ‘*in silico*-virus spiked’ datasets contain sequences from Bas-Congo virus (BASV), a novel rhabdovirus associated with hemorrhagic fever cases in central Africa (28). In *silico*-generated BASV sequences were computationally spiked at various read lengths and depths of coverage (Table 1) into a complex *in silico* metagenomic background consisting of 10 million human reads, 2.5 million bacterial reads and 0.5 million viruses, generating sets A through J. The *in silico* background reads were generated from the National Center for Biotechnology Information (NCBI) hg19, bacterial RefSeq and viral RefSeq databases, as described previously (1). In addition, two of the datasets contained *in silico* BASV reads that were computationally spiked into human metagenomic background NGS datasets generated from nasal swabs from patients with respiratory infection (*n* = 3 845 484 reads) and stool samples from patients with diarrheal disease (*n* = 9 652 958 reads), respectively. The two human background NGS datasets were obtained by Illumina HiSeq sequencing and were constructed as previously described (1). All *in silico* reads were paired-end reads and generated at a 2% error rate using the wgsim program in the SAMtools software package (29).

**Table 1.** BASV sequences representing varying degree of read length and coverage were spiked into synthetic background (setA to setJ) or real metagenomic background (Nasal, Stool) to create ‘*in silico*-virus spiked’ datasets

Dataset	Read Length	BASV Coverage	Background
setA	100	200	10M human + 2.5M bact + 0.5M viral
setB	100	20	10M human + 2.5M bact + 0.5M viral
setC	100	10	10M human + 2.5M bact + 0.5M viral
setD	100	4	10M human + 2.5M bact + 0.5M viral
setE	100	2	10M human + 2.5M bact + 0.5M viral
setF	600	60	10M human + 2.5M bact + 0.5M viral
setG	300	30	10M human + 2.5M bact + 0.5M viral
setH	150	15	10M human + 2.5M bact + 0.5M viral
setI	75	7.5	10M human + 2.5M bact + 0.5M viral
setJ	50	5	10M human + 2.5M bact + 0.5M viral
Nasal	100	10	3.8M nasopharyngeal swab sample
Stool	100	10	9.6M stool background containing a norovirus

The ‘pooled virus standard’ dataset corresponds to a biological reagent provided by the National Institute for Biological Standards and Control (NIBSC). This reagent is assembled from clinical specimens and egg- and cell-cultured passaged viruses and consists of a pool of 25 human viral pathogens from two DNA and seven RNA viral families, including adenovirus 2 and 41, herpesviruses 1–5, rotavirus A, astrovirus, norovirus GI and GII, sapovirus C12, coronavirus 229E, parechovirus 3, rhinovirus A39, coxsackievirus B4, influenza viruses A(H1N1), A(H3N2) and B, human metapneumovirus, respiratory syncytial virus and parainfluenzaviruses 1–4. The genome sizes of the reagent viruses ranged from ∼6 to ∼234 kb. The NGS dataset generated from Illumina MiSeq sequencing of the reagent contained ∼20 million raw 250 base pair (bp) paired-end reads.

The eight ‘human/animal pathogen’ datasets include a variety of pathogens of different types and genome sizes sequenced using the Illumina MiSeq or HiSeq platform from human and animal metagenomic samples (Table 2). Datasets I-IV contain NGS reads from four selected viral metagenomic libraries (human blood, human stool, animal tissue and animal stool) generated using the Nextera XT kit and sequenced as 250 bp Paired-end reads on the Illumina MiSeq platform (30). The four datasets contain 0.28–1.37 million raw NGS reads each and include sequences representing four viral genomes. Dataset V (virus) was generated from pooled pediatric diarrheal stool (1,31) and included sequences representing two viral genomes. Dataset VI (bacteria) was generated from a plasma sample from a patient from Africa with typhoid fever from *Salmonella typhi* bacteremia generated using a Truseq adapted method as described previously (*n* = 16 540 336 reads) (1). In addition, to compare the performance of the various *de novo* assembly approaches in traditional assembly of bacterial and eukaryotic pathogens from pure cultures, we also analyzed NGS datasets corresponding to cultured isolates of *Staphylococcus aureus* prepared using a Truseq DNA library preparation kit (Dataset VII, *n* = 1 million reads) and *Naegleria fowleri* (32) (Dataset VIII, *n* = 10 million reads), a parasitic amoeba that causes primary amebic meningoencephalitis.

**Table 2.** Eight datasets (‘human/animal pathogens’) containing at least nine pathogens with known genomic sequences represent various pathogen type, genome size, sample background and sequencing output that were encountered in real world metagenome and clinical applications using NGS

Dataset	Target genome	Read length	#reads	Genome type	Genome size	Description
I	*Feline sakobuvirus* (NC_022802)	250 + 250	1.37M	ssRNA virus	7059 (complete polyprotein)	Animal feces
II	*Unclassified phage 8L3*	250 + 250	1.28M	dsDNA virus	96429	Human feces
III	*Parvovirus B19*	250 + 250	0.28M	ssDNA virus	4876 (near complete genome)	Human blood
IV	*Enhydra lutris papillomavirus 1* (NC_023873)	250 + 250	0.34M	dsDNA virus	8109	Animal tissue
V	*Human parechovirus* (KJ152442)	75 + 75	12.4M	ssRNA virus	7217	Diarrheal pool of 8 individuals
V	*Human sapovirus* (AY646853)	75 + 75	12.4M	ssRNA virus	7429	Diarrheal pool of 8 individuals
VI	*Salmonella typhi* (CP002099)	100 + 100	16.5M	Bacterium	4791958	Plasma from patient with acute hemorrhagic fever
VII	*Staphylococcus aureus* (HF937103)	100 + 100	1.0M	Bacterium	2864125	Bacterial genome, pure culture
VIII	*Naegleria fowleri* mitochondrion (NC_021104)	100 + 100	10M	Eukaryote mitochondrion	49531	Isolation from the cerebrospinal fluid (CSF) of a patient

### Preprocessing

Raw reads obtained from Illumina sequencing were preprocessed before assembly as follows. Human host reads were subtracted by mapping the reads with human reference genome hg19 using bowtie2 (33). Reads that were identical from nucleotide positions 5–45 were considered clonal reads and only one random copy of clonal reads was retained. The other clonal sequences were replaced with sequence ‘A’ as a place holder; thus the original order of the paired-end files was preserved. A paired-end sequence record was removed only if both ends were replaced. Low-quality sequences were trimmed using a Phred quality score 10 as the threshold. Adaptor and primer sequences were trimmed using the BLAST-based VecScreen at default parameters (34).

### Ensemble assemblers

**Figure 1.**
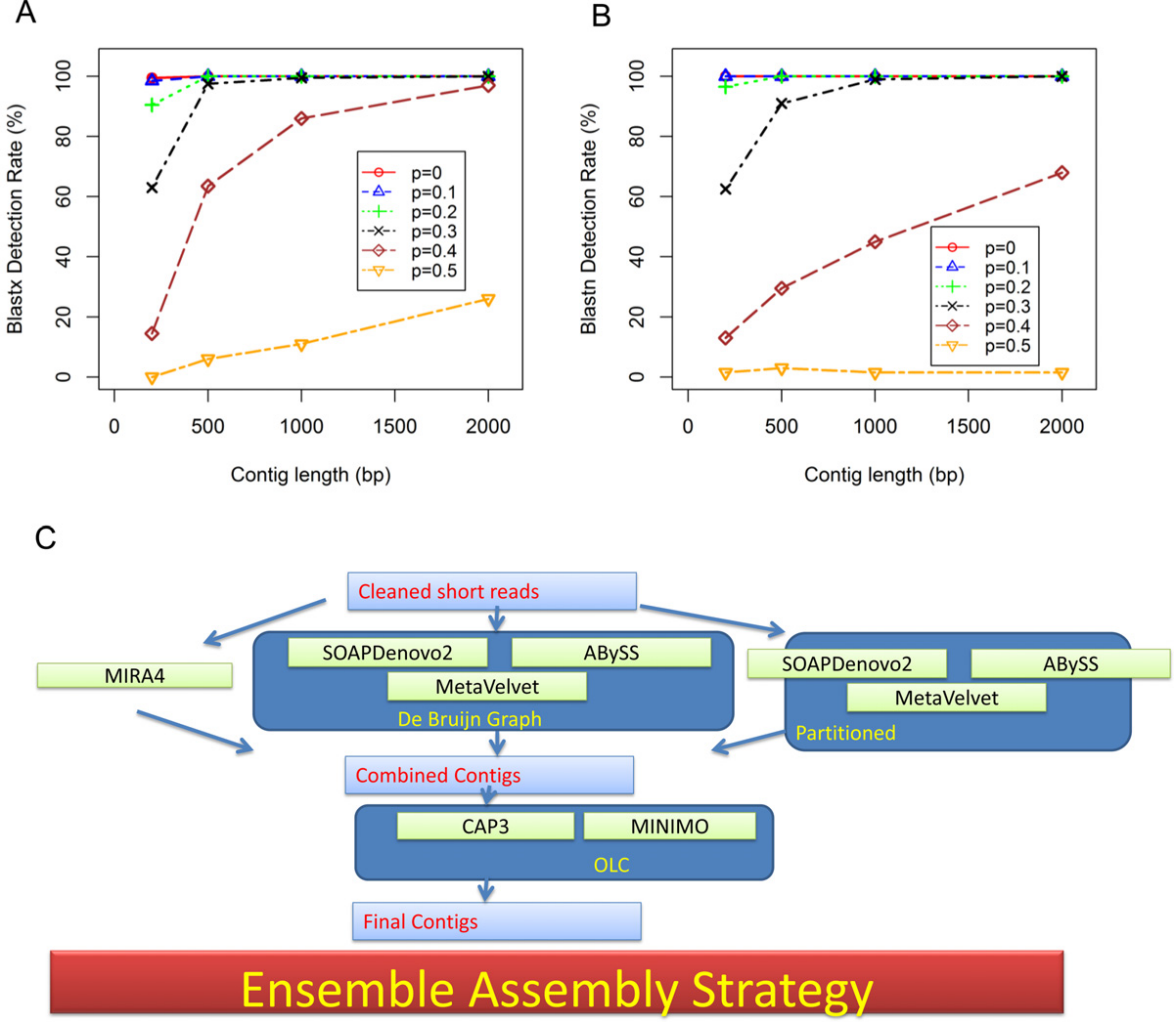
Motivation and design of the ensemble assembly strategy. (**A**) Detection rates using blastx at various sequence lengths and mutation rates. Sequences were randomly extracted from virus RefSeq at various lengths (200 bp, 500 bp, 1000 bp, 2000 bp). Each base were mutated at different probability (*P* = 0, 0.1, 0.2, 0.3, 0.4, 0.5) to simulate various degrees of divergence. (**B**) Detection rates using blastn at various sequence lengths and mutation rates. (**C**) The ensemble assembler that integrates DBG assemblers and OLC assemblers. The cleaned reads were first assembled individual DBG assemblers, partitioned assemblers or Mira4. The output of the first step is combined, length filtered and feed into the OLC assemblers for final assembly. The choice of individual assemblers as components can generate a number of ensemble assembly strategies.

As a proof of concept that longer viral contigs can be better detected, sequences of various lengths (200, 500, 1000 and 2000 bp) were extracted from Virus RefSeq (Release 61) and mutated at various probabilities (0, 0.1, 0.2, 0.3, 0.4, 0.5) for each base. We then applied blastx and blastn with *E*-value 0.01 as cutoff on the simulated contigs against Virus RefSeq protein or nucleotide database. Figure 1A shows that longer contigs clearly have a better chance to be detected by blastx, which is especially true for highly divergent contigs. Figure 1B shows the same pattern using blastn. However, amino acid-based search shows better detection rate for highly divergent organisms than nucleotide-based homology search.

We sampled commonly used assemblers including SOAPdenovo2_r240 (S), ABySS_1.3.7 (A), MetaVelvet_1.2.10 (V), OLC assemblers including Celera_wgs-8.1 (W), Cap3 (C) and Minimo_amos-3.1.0 (O) and other assemblers including Omega_v1.0.2 (G), Mira_4.0.2 (M),MaSuRCA-2.2.0 (X), IDBA-UD_1.1.1(I) and Trinity_r20140717 (T). Partitioning was performed by randomly splitting original sequences into chunks of 100K reads each. The *de novo* assembly was then performed on each chunk separately. Resultant contigs from each chunk were then combined as output. We use ‘s’, ‘a’ and ‘v’, to represent partitioned assembly for SOAPdenovo2, ABySS and MetaVelvet respectively.

Figure 1C outlines the ensemble assembly strategy, which can be viewed as a 3-step process. The first step is to perform S, A, V and their corresponding partitioned versions s, a, v individually. The second step is to combine one or more output contigs from the first step and use a length filter (e.g. 300 bp used here) to only retain longer contigs above a certain threshold. The purpose of the length filtering is to eliminate smaller contig fragments that can significantly slow down the final step. The final step is to apply O or C on the combined contigs to generate final contigs. We also tested whether using T or M at the second assembly step can produce performance advantages, although T and M are technically not OLC assemblers. The ensemble assembly algorithms are labeled by their components. For example, SC represents S followed by C; SAVaO represents first combining the outputs of S, A, V and a, and then applying O as the last step.

The main conventional quality metric for *de novo* assembly is the contig size. We initially evaluated the commonly applied N50 or N95 metrics (data not shown), but decided that they are not applicable here, because N50 and N95 can be significantly skewed if the assembly output contains a large number of small fragments, as is frequently the case for metagenomic data. Furthermore, N50 and N95 do not specifically address assembly of small target genomes within a complex metagenomic background (a ‘needle-in-a-haystack’ problem). Direct measurements of the sizes of contigs aligning to known target microbial genomes and of the degree of misassembly are more relevant for metagenomic samples. Thus, we defined three alignment-based parameters as metrics for the efficiency of *de novo* assembly of target genomes: (1) the ‘Max Aligned Contig Region (MACR)’ or ‘Max Aligned Contig Region Percentage (MACRP)’, the size (bp) or percent coverage (%) of the target genome achieved by the single longest local alignment between any contig and that genome; (2) the ‘C1000’, the size (bp) or percent coverage (%) of the target genome covered by alignments 1000 bp or larger; and (3) the ‘chimera index’, the percentage of unaligning regions within all contigs that align to the target genome. Specifically, for a given target genome of size R, the definitions of MACR, MACRP, C1000 and chimera index are as follows:

CT: the full set of contigs that can be aligned to the target genome
AL: all aligned regions in CT
AL1000: all aligned regions in CT that are 1000 bp or greater
UA: all unaligned regions in CT
M: summed length of AL (bp)
N: summed length of UA (bp)
C1000: union of AL1000 by merging the overlapped regions
MACR (bp) = max (AL)
MACRP (%) = max (AL)/R × 100%
Chimera Index (%) = N/(M+N) × 100%

MACR and MACRP measure single largest alignment size, C1000 measures collectively the size of large alignments and chimera index measures the percentage of incorrect contig formation. Supplementary Figure S1 shows an example how these metrics were computed.

Determination of the exact alignment between contigs and target genome was performed using the stringent alignment program MegaBLAST (34) at default parameters. We also recorded timing performance metrics for each run. All assembly runs were executed on identical Intel® 8-core Xeon servers each with 32 GB memory and 1 TeraBytes direct attached storage. The S, A, V, M, W, T, X, s, a, v assemblies were all executed using 8-threads, whereas O and C only support single-thread processing.

### Composite performance metric and ranking of assemblers

We generated comparative performance metrics for the different assemblers by normalizing each metric into a range of 0–5, with larger values representing better performance. For example, given the C1000 metric, the corresponding normalized ratio, denoted as C1000_NR_ for a specific target genome, can be defined as:

\begin{eqnarray*} &&{\rm C}1000_{\rm NR} = \nonumber \\ &&5 \times {\rm C}1000/{\rm max}({\rm C}1000\;{\rm of}\;{\rm all}\;{\rm assemblers}). \end{eqnarray*}

Similarly, we define

\begin{eqnarray*} &&{\rm MACR}_{\rm NR} = \nonumber \\ &&5 \times {\rm MACR}/{\rm max}({\rm MACR}\;{\rm of}\;{\rm all}\;{\rm assemblers}), \end{eqnarray*}

\begin{eqnarray*} &&{\rm Accuracy}_{\rm NR} = \nonumber \\ &&5 \times (1 - {\rm ChimeraIndex}/{\rm max}({\rm ChimeraIndex}\;{\rm of}\;{\rm all}\;{\rm assemblers})), \end{eqnarray*}

\begin{eqnarray*} &&{\rm Speed}_{\rm NR} = \nonumber \\ &&5 \times (1 - {\rm Time}/{\rm max}({\rm Time}\;{\rm of}\;{\rm all}\;{\rm assemblers})). \end{eqnarray*}

The composite performance metric (CPM) is the weighted average of four metrics about contig qualities. We assign equal weights to alignment size and accuracy:

\begin{eqnarray*} &&{\rm CPM} = 0.25 \times ({\rm MACR}_{\rm NR} + {\rm C}1000_{\rm NR})+ \nonumber \\ &&0.5 \times {\rm Accuracy}_{\rm NR} \end{eqnarray*}

Based on CPMs calculated for each assembler and target genome, we can rank the assemblers based on the average CPMs across all target genomes.

## RESULTS

### Determination of optimal *k*-mer size for DBG assemblers

**Figure 2.**
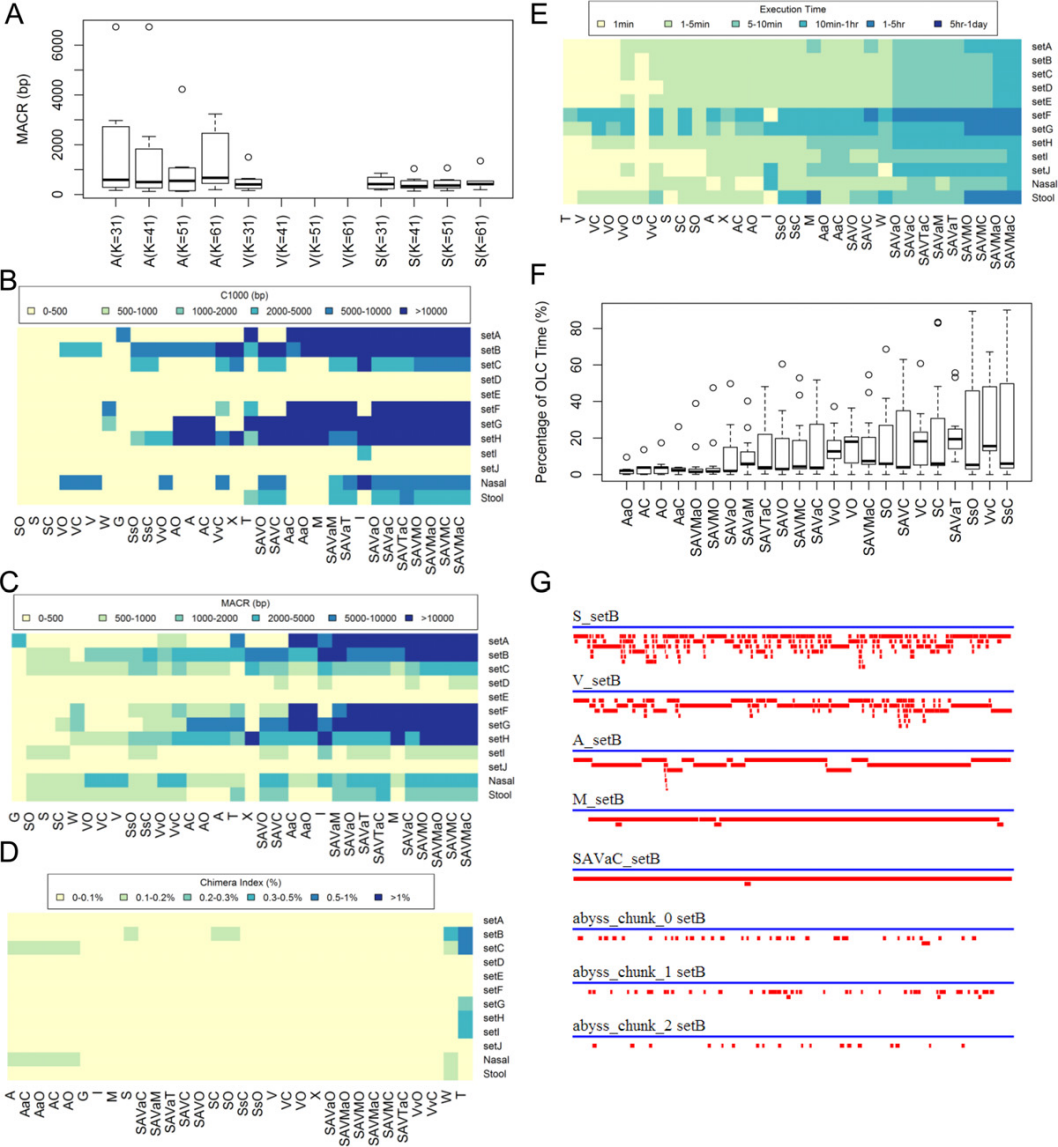
Comparison of different assembly strategies using the *in silico*-virus spiked BASV datasets. (**A**) Comparing MACR for different *K*-mer size using A, V and S on the *in silico*-virus spiked BASV dataset (SetA-SetJ). Note that V only supports *K* ≤ 31. The bottom and top of the box are always the first and third quartiles, and the band inside the box is the median; (**B**) C1000 distribution for each assembler; (**C**) MACR for each dataset; (**D**) chimera index; (**E**) execution time; (**F**) percentage of execution time spent on OLC final assembly; and (**G**) contig formation for setB: the blue line represents the 12 648 bp BASV genome. The red lines are contigs that are aligned to the BASV genome. The assemblers of Figure B, D, E and F were ordered by average values. Individual assemblers A, S, V, M, T, W were executed using eight threads and O and C were executed using single thread.

Most DBG assemblers require that a *k*-mer size be provided as a configurable parameter. As the choice of an optimal *k*-mer value is not clear with metagenome assembly, we tested S and A using the ‘*in silico*-virus spiked’ datasets at increasing *k*-mer values of 31, 41, 51 and 61 (V does not support *k*-mer values >31) (Figure 2A). *K*-mer values ranging from 31 to 61 have previously been shown to be useful for DBG assemblers, whereas shorter *k*-mer values below 31 seem to generate shorter contigs (35). Using the ‘*in silico*-virus spiked’ dataset, A performed better than S or V. For the S or A algorithms, no significant differences were observed by varying the *k*-mer values from 31 to 61 ( *P* > 0.05, Kraskal–Wallis test). Since *k*-mer values must be smaller than the read length, we chose *k* = 31 as providing the greatest flexibility in analysis of very short reads and keeping the parameter constant for comparative benchmarking of the S, A and V algorithms. It should be noted that the choice of optimal *k*-mer depends on the data being applied. Here we use *k* = 31 for this study, but it may not be optimal on other datasets.

### Using various assembly strategies to test ‘*in silico*-virus spiked’ datasets

With the *in silico*-virus spiked datasets, we tested a number of different assembly strategies: (i) individual assembly with S, A, V, M, G, T, X and W alone; (ii) two-step assemblies with SC, AC, VC, SO, AO or VO; (iii) partitioning schemes with AaC, SsC, VvC, AaO and SsO, (iv) combining multiple DBG assemblers with SAVC and SAVO; (v) combining multiple DBG assemblers and partitioning with SAVaC and SAVaO; (vi) adding M and T to other assembly schemes with SAVMC, SAVMO, SAVMaC, SAVMaO and SAVTaC; and (vii) using M or T at the second step as a replacement for OLC assembly rather than at the first step with SAVaM or SAVaT.

To calculate the MACRP, C1000 and chimera index metrics, contigs were aligned to the target *in* *silico*-spiked viral genome, BASV, using MegaBLAST (Figure 2B–G). Here we chose to use BASV for our spiked viral genome because it is a novel, highly divergent virus with a uniquely identifiable sequence and has no known close viral relatives (28). Widely different results were observed across the various assembly strategies after analysis of the *in silico*-virus spiked datasets using different read lengths and depths of BASV coverage (Figure 2B, sets A–J) and two clinical sample backgrounds (Figure 2B, ‘Nasal’ and ‘Stool’). Performance improvements were observed with the inclusion of M or partitioning scheme ‘a’, with the best ensemble combinations assembling the nearly complete genome (∼12 kb) in a single contig that is significantly larger than what was achieved with DBG assemblers alone (Figure 2C). The individual DBG assemblers A, S, V, T and OLC assembler W all generated typically poor-sized contigs even in high-coverage datasets (Figure 2C, setA, 200x coverage; setF, 60x coverage). M was the only individual assembler found to produce good-sized contigs in certain cases (setA, set F–H). Many simple combinations, such as SC, AO and VC, did not result in an improvement in overall performance.

Not surprisingly, increasing coverage resulted in better assembly in terms of greater MACR for all assembly methods (Figure 2C). With setA, setB, setF and setG, all containing *in silico* BASV reads at >20× coverage, nearly 100% of the genome could be obtained for some of the assembly combinations. With datasets at <15× coverage, none of the methods produced contigs >5 kb in length. Notably, SsC, SsO, VvO and VvC did not perform nearly as well as AaO and AaC, suggesting that partitioning is most advantageous when using A. Using M as the final assembler (SAVaM) appeared to generate slightly worse contigs than using C (SAVaC), O (SAVaO) or T(SAVaT), although SAVaM was also slightly faster than the others (Figure 2C and E). Although the number and distribution of BASV ‘spike-in’ reads were identical for the nasal and stool datasets, *de novo* assembly of BASV in the nasal dataset was consistently better than in the stool dataset, indicating that a less complex background (as is the case for respiratory secretions relative to stool) is a favorable factor for assembly. All of the assembly combinations yielded very low levels of misassemblies on analysis of the *in silico*-spiked virus datasets, with a chimera index consistently <0.15%, except for a few datasets analyzed using W and T in which the chimera index was as high as 0.8% (Figure 2D).

When comparing the timing for each assembler (Figure 2E), we found that assemblers A, S, V and T were relatively fast, typically finishing within seconds to minutes. As expected, OLC assembler W took longer to complete than A, S and V. Among all individual assemblers, M took the longest to run on average, taking anywhere from a few minutes to a few hours to finish. Thus, any ensemble assembly with M as a component took significantly longer to run.

The total run-time corresponding to ensemble strategies is the sum of the run-time for each individual component assembly: (i) an individual assembly (A, S, V, M and T), (ii) a partitioned assembly (a, s, v) and (iii) the OLC step (C, O, M and T). We executed the components of ensemble assemblers sequentially on a single server and recorded the total run-time for all datasets (Figure 2E). In practice, however, the timing performance of an ensemble assembly method can be improved by executing the first-step assemblies (A, S, V, M, a, s, v) in parallel, provided that multiple servers are available. To test whether the second OLC assembly step is an execution bottleneck, the relative percentage time spent on the second assembly step was calculated (Figure 2F). In most cases, the second OLC step did not appear to be a significant bottleneck (<∼20% of the execution step). Nevertheless, in the worse-case scenario, more than 80% of the execution step was spent on the second OLC assembly step.

Mapping of the contigs produced by the S, A, V, M, SAVaC and three different partitions on the BASV genome revealed that distinct albeit short contigs are produced by the three individual DBG assemblers and by partitioning (Figure 2G). When combined, these distinct contigs may help to form overlaps, fill gaps and facilitate the construction of much larger contigs, suggesting the utility of combining DBG assemblers with partitioning for the first assembly step.

### Using various assembly strategies to test ‘pooled virus standard’ dataset

**Figure 3.**
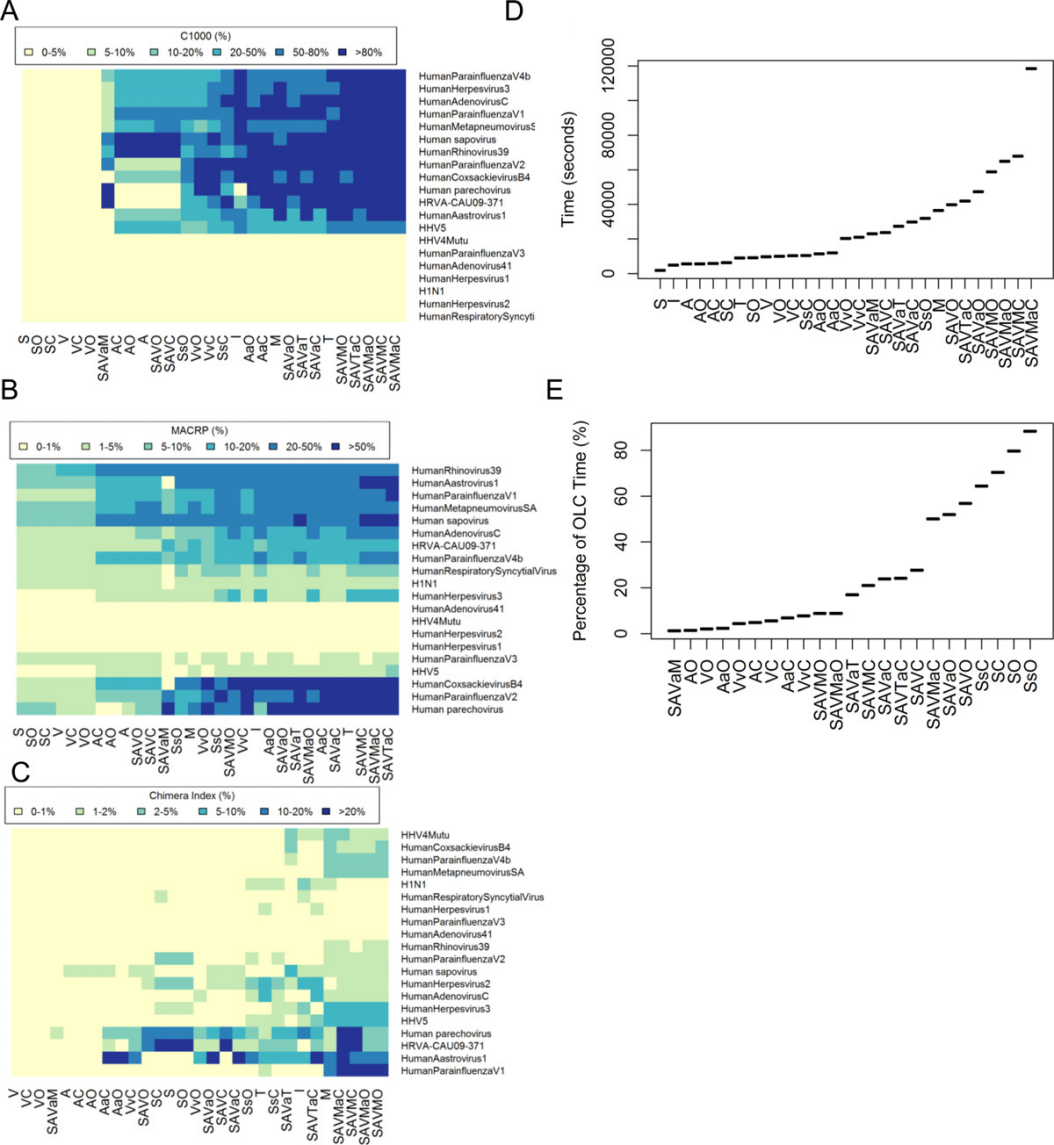
Comparison of different assembly strategies using the ‘pooled virus standard’ dataset: (**A**) C1000 for each assembler; (**B**) MACRP for each assembler; (**C**) chimera index as a heatmap; (**D**) execution time; and (**E**) percentage of execution time on the OLC step. All boxplot figures were ordered by average values on the y-axis.

When benchmarking the performance of various assembly strategies with the ‘pooled virus standard’ dataset, we observed that the ranking of assemblers by C1000 and MACRP was similar to those in the ‘*in silico*-virus spiked’ datasets (Figure 3A and B). The assembler T, however, performs surprisingly well for this dataset in terms of MACR and C1000, unlike what was observed in the ‘*in silico*-virus spiked’ datasets (Figure 2B and C). The best assembly strategies, except T individually, were all found to be ensemble assembly combinations with partitioning or including M or both. One of the best ensemble assembly methods, SAVaC, produced a median MACRP of 17.5%, a 7-fold improvement over S alone (2.4%). Heat map analysis of the MACPR parameter for the top 20 viruses with contig alignments (Figure 3B) showed the superiority of the ensemble assembly strategies SAVaC and AaO (>20% of the target genome assembled for eight viruses) over the individual DBG assemblers (>20% of the target genome assembled for only two viruses). M, T and combinations with M or T showed an elevated level of chimeric assembly as compared to strategies using other assemblers, in which the chimeras index for most viruses was <1% (Figure 3C). Timing analysis revealed that the ensemble combination AaO was almost two-fold faster than SAVaC, while maintaining almost equivalent contig quality for this dataset (Figure 3A and B). However, the three slowest ensemble assembly strategies were also found to use O, suggesting that O may not scale well with the larger input sizes generated using multiple DBG assembly combinations (SAVO and SAVaO) or inefficient DBG assemblers (SsO) (Figure 3D). For this large dataset with 20 million reads, the percentage time spent on OLC step is 29% for SAVaC and 4% for AaO respectively (Figure 3E), suggesting that the ensemble assemblers are relatively efficient in their handling of larger datasets.

### Using various assembly strategies to test the ‘human/animal pathogen’ datasets

**Figure 4.**
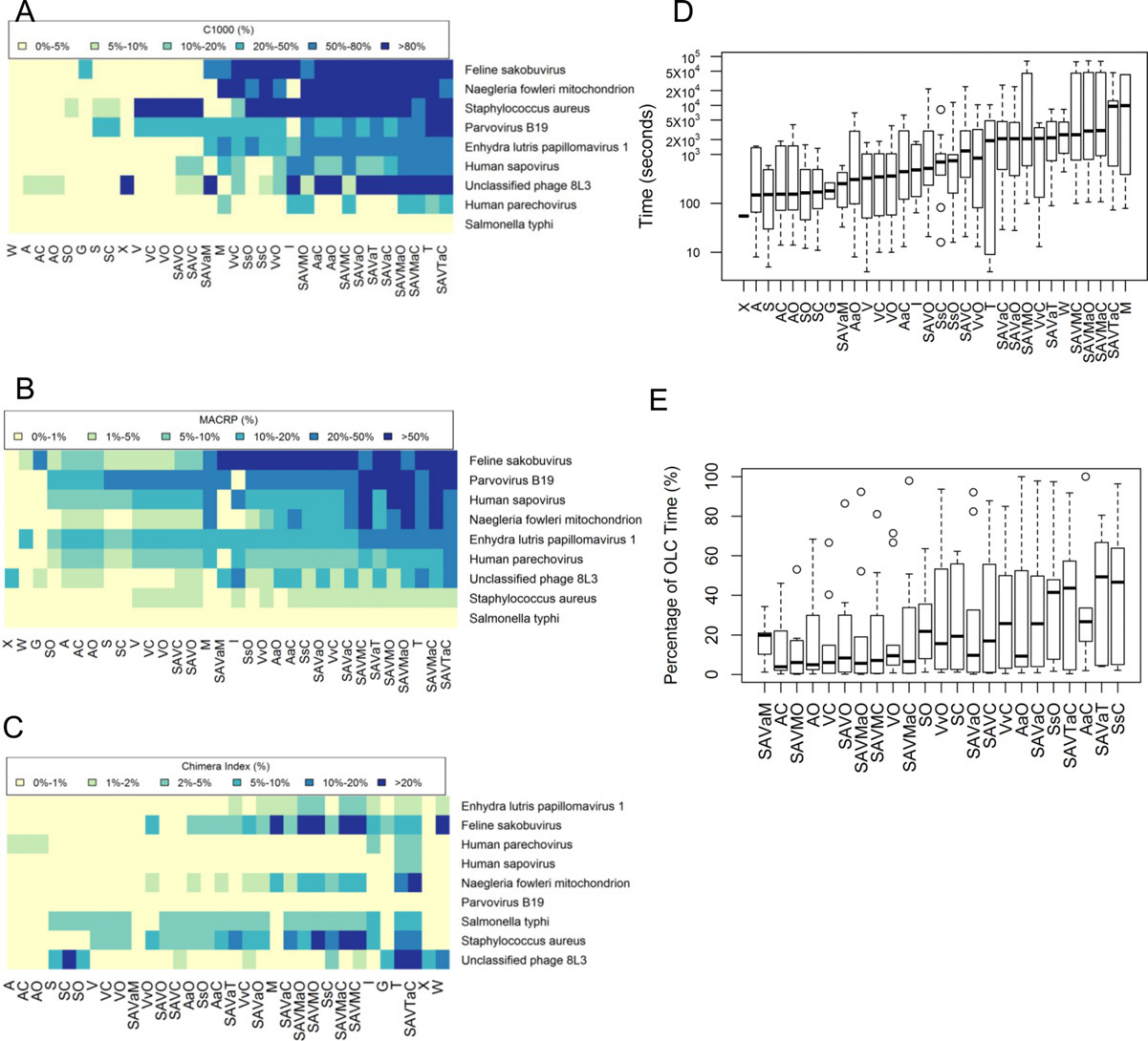
Comparison of different assembly strategies using the ‘human/animal pathogens’ eight pathogen datasets: (**A**) C1000 distribution; (**B**) MACRP distribution; (**C**) chimera index distribution; (**D**) execution time; and (**E**) percentage of execution time on the OLC step. All boxplot figures were ordered by average values on the y-axis.

To extensively test these assembly strategies across multiple datasets, eight metagenomic ‘human/animal pathogen’ datasets containing nine target genomes (feline sakobuvirus (NC_022802), unclassified phage 8L3, parvovirus B19, *Enhydra lutris* papillomavirus 1 (NC_023873), human parechovirus (KJ152442), human sapovirus (AY646853), *S. typhi* (CP002099), *S. aureus* (HF937103) and *N. fowleri* mitochondrion (NC_021104)) were tested using various assembly strategies (Figure 4). Similar to the C1000 and MACP distributions in the ‘*in silico*-virus spiked’ datasets and the ‘pooled virus standard’ dataset (Figures 2 and 3), the strategies that employ M or T generated the largest contig alignments (Figure 4A and B). However, these strategies also resulted in the highest level of misassemblies, with significant increases in the average chimera index to 5% or higher (Figure 4C). Ensemble assembly strategies without M or T, such as SAVaC, showed much lower levels of misassembly, with a chimera index typically under 5%. Similar to the other datasets, the assembly performance of the individual DBG assemblers was limited, with only 3–10% of target genomes being successfully *de novo* assembled versus >20% of target genomes for the top ensemble strategies. For example, heat map analysis of multiple microbial genome targets confirmed the superiority of ensemble strategies such as SAVaC and SAVTaC (Figure 4A and B). Notably, SAVaC achieved a C1000 metric of >80% in four of nine target genomes, versus only zero or one of nine genomes for the S, A, V and M algorithms run individually. The timing data (Figure 4D) showed that using M individually or as a component in the first assembly takes a significantly longer time to execute than other methods. While the timing differences were not as pronounced as in the ‘pooled virus standard’ dataset, the AaO strategy, as well as the related AaC and VvO strategies, were overall faster than SAVaC but resulted in the generation of shorter contigs. The percentage of time spent in the OLC step was also found to be acceptable, with average case below 50%, in these very diverse metagenomic datasets (Figure 4E).

### Normalized and composite performance metrics

**Figure 5.**
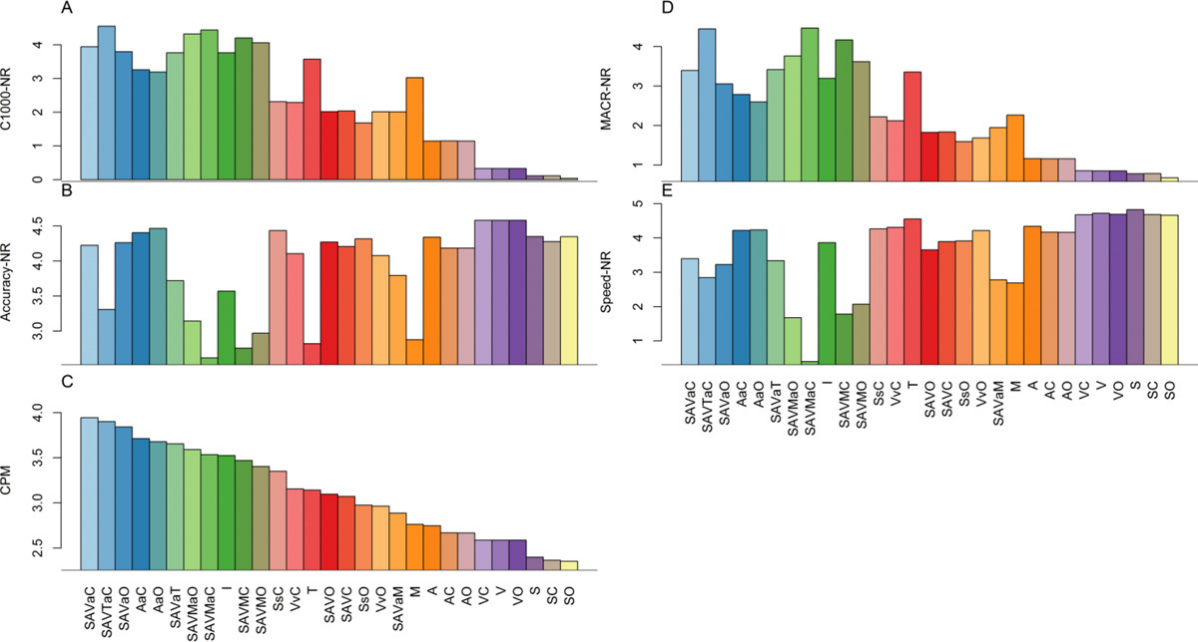
Normalized measures and CPMs averaged over all target genomes for each assembler: (**A**) C1000_NR_ distribution; (**B**) Accuracy_NR_ distribution; (**C**) CPM distribution; (**D**) MACR_NR_ distribution; and (**E**) Speed_NR_ distribution. These measures are in the range of 0–5 with higher values representing better performance. Numbers in parentheses are numbers of target genomes evaluated for each method. Genomes that no assembler could generate MACR > 1 kb were excluded in the calculation. Note that certain assemblers such as G, W, X and SAVaM failed to finish in many of the datasets due to software issues.

**Figure 6.**
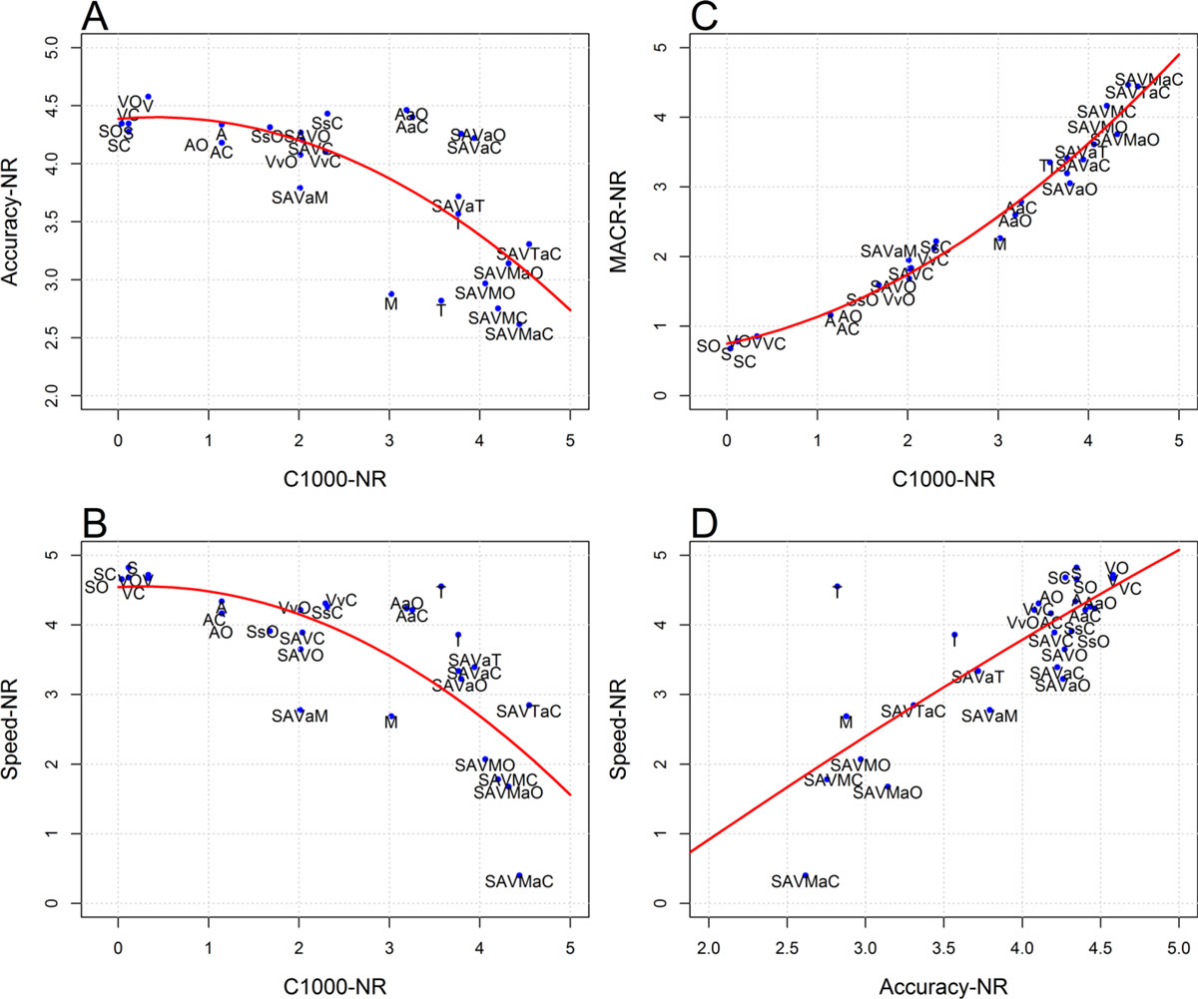
Relationship of normalized performance metrics: (**A**) Accuracy_NR_ versus C1000_NR_; (**B**) Speed_NR_ versus C1000_NR_; (**C**) MACR_NR_ versus C1000_NR_; and (**D**) Speed_NR_ versus Accuracy_NR_. In all of the four plots, upper right corner represent ideal performance. The quadratic regression curves were computed without including the method W.

To summarize the results from the three datasets, we calculated the average normalized performance metrics MACR_NR_, C1000_NR_, Accuracy_NR_, Speed_NR_ and composite performance metrics CPM across target genomes in all datasets for each assembler (Figure 5). For individual assemblers, I and T clearly produced the best C1000_NR_ among individual assemblers; whereas A, S V W, G produced poor sized contigs (Figure 5A). Ensemble strategies including partitioning or M or T produced the best C1000_NR_. The MACR_NR_ is highly correlated with C1000_NR_ (Figure 5D). T, M, I and ensemble methods using them as a component, however, are among the worst assembler in Accuracy_NR_ (Figure 5B). M and methods with M as a component, are the slowest (Figure 5E), indicating they are not suitable for time-critical diagnosis applications. According to CPM which measures overall contig qualities, the highest ranked assemblers were SAVaC, SAVTaC, SAVaO (Figure 5C). The best individual assembler is I but its CPM is still significantly lower than the best ensemble assemblers. SAVTaC achieved very high C1000_NR_ and MACR_NR_, but its Accuracy_NR_ and Speed_NR_ were below average. SAVaC and SAVaO achieved better Accuracy_NR_ and competitive C1000_NR_ and MACR_NR_. Figure 6 shows the relationship of the normalized measures. Figure 6A shows that assemblers fall in a curved belt, indicating a reciprocal relationship between Accuracy_NR_ and C1000_NR_. Ensemble methods with M or T as a component in the first assembly had the largest C1000_NR_ and poorest Accuracy_NR_, suggesting these assemblers may be overly aggressive in assembly of larger contigs. On the side of the curve were DBG assemblers which may be overly conservative in extending contigs. SAVaO and SAVaC are closest to the ideal upper right corner, indicating they achieved balance overall performance. Figure 6B shows that C1000_NR_ is negatively correlated with Speed_NR_. C1000_NR_ and MACR_NR_ are both contig size measures and they are closely correlated (Figure 6C). Figure 6D shows positive correlation between Accuracy_NR_ and Speed_NR_, indicating faster assemblers are generally conservative and thus generate lower level of chimeric contigs.

Table 3 compares MACR for DBG assemblers A, V, S with our ensemble assemblers, AaO and SAVaC across all datasets. It was observed that SAVaC produced MACR that are 7.3, 11.9 and 13.6 fold as large as those produced by A, V and S. SAVaC also produced contigs that are 60% larger than AaO on average. Tables 4 and 5 show detailed descriptions and command-line parameters/configurations for preprocessing and running *de novo* assemblers respectively.

**Table 3.** MACR (bp) of ensemble assemblers (SAVaC and AaO) and individual assemblers

Dataset:target genome	A	V	S	AaO	SAVaC
setA:BASV	235(54)	158(80)	183(69)	12 555(1)	**12 617(1)**
setB:BASV	2969(4.2)	1501(8.3)	796(16)	3439(3.6)	**12 522(1)**
setC:BASV	758(2.2)	645(2.6)	857(2)	758(2.2)	**1694(1)**
setF:BASV	776(16)	200(63)	234(54)	**12 538**(1)	**12 538(1)**
setG:BASV	6730(1.8)	269(46)	233(53)	12 396(1)	**12 445(1)**
setH:BASV	2487(1.7)	573(7.5)	448(9.6)	2487(1.7)	**4304(1)**
Stool:BASV	436(3.7)	718(2.2)	857(1.9)	436(3.7)	**1595(1)**
Nasal:BASV	758(3.5)	2667(1)	857(3.1)	758(3.5)	**2667(1)**
Pooled:HHV4Mutu	991(1.3)	804(1.6)	804(1.6)	991(1.3)	**1291(1)**
Pooled:HHV5	3040(1.4)	944(4.6)	923(4.7)	**4350**(1)	**4350(1)**
Pooled:HRVA-CAU09–371	229(14)	299(11)	301(11)	2572(1.3)	**3302(1)**
Pooled:Human parechovirus	89(80)	283(25)	388(18)	7155(1)	**7155(1)**
Pooled:Human sapovirus	3491(1)	568(6.4)	512(7.1)	**3640**(1)	**3640(1)**
Pooled:HumanAastrovirus1	1246(2.6)	464(6.9)	400(8)	2747(1.2)	**3186(1)**
Pooled:HumanAdenovirusC	1582(4.3)	899(7.5)	586(12)	4565(1.5)	**6744(1)**
Pooled:HumanCoxsackievirusB4	1349(5)	251(27)	317(21)	6664(1)	**6800(1)**
Pooled:HumanHerpesvirus3	2425(1.8)	776(5.7)	480(9.3)	**5599**(0.79)	4451(1)
Pooled:HumanMetapneumovirusSA	2259(1.6)	877(4.1)	878(4)	3494(1)	**3555(1)**
Pooled:HumanParainfluenzaV1	2502(2.8)	710(9.8)	681(10)	**6924**(1)	**6924(1)**
Pooled:HumanParainfluenzaV2	1065(13)	365(37)	296(46)	4095(3.3)	**13 488(1)**
Pooled:HumanParainfluenzaV4b	2624(1)	781(3.4)	751(3.5)	**2624**(1)	**2624(1)**
Pooled:HumanRhinovirus39	2768(1.1)	769(4)	643(4.8)	2772(1.1)	**3112(1)**
Pathogens:*Enhydra lutris* papillomavirus 1	893(1.7)	890(1.7)	654(2.4)	**1539(1)**	**1539(1)**
Pathogens:Feline sakobuvirus	476(12)	339(17)	311(18)	**5596(1)**	**5596(1)**
Pathogens:Human sapovirus	654(3.3)	801(2.7)	483(4.5)	1009(2.2)	**2182(1)**
Pathogens:*Naegleria fowleri* mitochondrion	501(20)	841(12)	169(60)	8235(1.2)	**10 158(1)**
Pathogens:Parvovirus B19	793(2.7)	2162(1)	2162(1)	1322(1.6)	**2162(1)**
Pathogens:*Salmonella typhi*	DNF	1000(2.2)	2227(1)	1324(1.7)	**2229(1)**
Pathogens:*Staphylococcus aureus*	DNF	40 417(2.5)	3617(28)	17 606(5.7)	**100 476(1)**
Pathogens:Unclassified phage 8L3	2365(5.9)	247(57)	301(47)	13 809(1)	**14061(1)**
Average MACR (fold versus SAVaC)	**1327(7.3)**	**1645(11.9)**	**647(13.6)**	**3954(1.6)**	**6859(1)**
*P*-value	**3.8 × 10^−7^**	**1.2 × 10^−7^**	**8.1 × 10^−8^**	**6.7 × 10^−6^**	

Numbers represent MACR (bp) and numbers in parenthesis represent MACR fold differences compared with SAVaC. Bold numbers represent largest MACR for each row. The paired Mann–Whitney U tests were performed to test whether an assembler produced MACR that are significantly lower than SAVaC. DNF: Did not finish.

**Table 4.** Preprocessing procedures and parameters for metagenomic datasets

Pre-processing	Example command	Description/configuration
1. Host sequence subtraction	bowtie2 –quiet –local –no-hd –reorder -p 8 -x human_bac_bowtie -U raw_read.fq.gz -S read.sam	Human host reads were subtracted by mapping the reads with human reference genome hg19 using bowtie2
2. Sequence De-duplication	dedup input1.fastq deduped2.fastq	Reads that were identical from nucleotide positions 5–45 were considered clonal reads, and only one random copy of clonal reads was retained. The other clonal sequences were replaced with sequence ‘A’ as a place holder; thus the original order of the paired-end files was preserved.
	dedup input1.fastq deduped2.fastq	
	(The program dedup is provided with the main program)	
3. Adaptor and quality trimming	blastn -task blastn -evalue 1 -gapopen 5 -gapextend 3 -penalty -5 -max_target_seqs 100 000 000 -outfmt 6 -query adaptor.fa -num_threads 8 -db reads.db -out reads.tab	Adaptor and primer sequences were trimmed using the BLAST-based VecScreen at default parameters. Low-quality sequences were trimmed using a Phred quality score 10 as the threshold.

**Table 5.** Parameters and configurations for *de novo* assembly experiments

Ensemble Assembler1.0	ensembleAssembly./config.txt	PE = 260 30 read1.fq read2.fq
	./ensemble.sh	NUM_THREADS = 8
		SOAP_KMER = 31
		ABYSS_KMER = 31
		METAVELVET_KMER = 31
		CON_LEN_DBG = 150
		CON_LEN_OLC = 300
		ASSEMBLY_MODE = VO, AaC, …
Cap3 (C)	cap3 input.fasta	
Minimo_amos-3.1.0 (O)	Minimo input.fasta -D FASTA_EXP = 1	
SOAPdenovo2_r240 (S)	SOAPdenovo-63mer all -K 31 -s read_soap.config -R -o read_soap	max_rd_len = 600
		[LIB]
		avg_ins = 260
		reverse_seq = 0
		asm_flags = 3
		rank = 1
		q1 = read1.fq
		q2 = read2.fq
ABySS_1.3.7 (A)	abyss-pe -C read_abyss name = read_abyss k = 31 in = ‘read1.fq read2.fq’	
MetaVelvet_1.2.10 (V)	velveth read_velvet 31 -shortPaired -fastq read1.fq read2.fq && velvetg read_velvet -exp_cov auto -ins_length 260 && meta-velvetg read_velvet -ins_length 260	
Mira_4.0.2 (M)	mira -t 8 read_mira.conf	project = setAHumBac
		parameters = -GE:not = 8 -DI:trt = /home/user/ -OUTPUT:rtd = yes
		job = genome,denovo,accurate
		readgroup = DataIlluminaPairedLib
		autopairing
		data = read1.fq read2.fq
		technology = solexa
IDBA-UD_1.1.1(I)	idba -r read.fa -o read_idba	
Omega_v1.0.2 (G)	omega -se 1 read.fq -l 60 -f read_omega	
Celera_wgs-8.1 (W)	fastqToCA -libraryname read_celera -technology none -mates read1.fq,read2.fq > read.frg	
	runCA -d read_celera -p read_celera read.frg	
MaSuRCA-2.2.0 (X)	masurca config.txt && assemble.sh	DATA
		PE = pe 260 30 read1.fq read2.fq
		END
		PARAMETERS
		GRAPH_KMER_SIZE = auto
		USE_LINKING_MATES = 1
		LIMIT_JUMP_COVERAGE = 60
		CA_PARAMETERS = ovlMerSize = 30 cgwErrorRate = 0.25 ovlMemory = 4GB
		KMER_COUNT_THRESHOLD = 1
		NUM_THREADS = 8
		JF_SIZE = 100000000
		DO_HOMOPOLYMER_TRIM = 0
		END
Trinity_r20140717 (T)	Trinity –seqType fq –JM 25G –output read_trinity –left read1.fq –right read2.fq –CPU 8	

## DISCUSSION

Here we introduced an ensemble strategy that sequentially integrates DBG and OLC assemblers and leverages the use of a partitioning approach for *de novo genome* assembly of pathogens in metagenomic NGS data. By benchmarking of the strategy using test datasets that include both *in silico*-generated and ‘real-life’ clinical and environmental metagenomic data, we demonstrated that ensemble assembly strategies produced accurate contigs that were significantly larger than those obtained by individual assemblers alone. Furthermore, the degree of misassembly for most of the ensemble strategies generally remained below 5%. Taken together, our data suggests that the best ensemble assembly strategy among those tested may be SAVaC (SoapDenovo2, ABySS, MetaVelvet, partitioned ABySS followed by Cap3), with AaO (Abyss, partitioned AbySS followed by Minimo) or AaC (Abyss, partitioned AbySS followed by Cap3) a reasonable alternative if fast execution time is of importance. Although the performance of some individual assemblers, such as M (Mira4) and T (Trinity) was comparable, the percentage of chimeric contigs was also high, and M (Mira4) was also computationally slow relative to the other assembly methods. IDBA-UD may be the best with respect to speed and contig alignment size among individual assemblers, despite its higher percentage of chimeras.

The poor performance of individual DBG assemblers in general for metagenome assembly can likely be attributed to (i) the use of *k*-mers instead of single reads which discards key layout and position information and (ii) the optimization of DBG methods for assembly of bacterial and eukaryotic genomes from pure cultures and not for metagenome assembly. Although the latest version of V (MetaVelvet/Velvet) was specifically developed for metagenomes by decomposing initial graphs into sub-graphs representing isolated species (16), it did not perform well individually in our testing. To our surprise, Celera (W), despite being an OLC assembler, generated poor, small contigs and the highest percentage of chimeric sequences. Another recently published assembler, G (Omega) (22), neither a DBG assembler nor an OLC assembler, did not perform well in the ‘*in silico*-virus spiked’ datasets (Figure 2). The MaSuRCA assembler (X) (19), which is a recent hybrid DBG and OLC assembler, did not generate large contigs as we would expected.

Because of the need to perform all potential pairwise alignments between reads and calculate overlaps (7), OLC assemblers are computationally impractical when applied directly to a large set of raw NGS sequences. Our ensemble strategy leverages fast DBG assemblers to quickly reduce the original NGS reads to a much smaller, non-redundant set of intermediate contigs. Our results suggest that partitioning is an indispensable part of the ensemble strategy. The use of a partitioning method produces additional distinct, albeit short contigs that can be used to fill gaps in coverage. The much slower OLC assemblers are used only in the final step to extend the contigs, thereby achieving much longer contigs than possible with DBG or OLC assemblers alone. Because the number of contigs is typically only 1–3% of the number of raw reads, the timing for the second OLC step is usually not a performance bottleneck. We note, however, that a second round of OLC assembly has the potential to introduce additional misassemblies, and indeed, the misassembly level of the ensemble strategies, although remaining <5% in general, is still higher than that of most of the individual DBG assemblers.

For metagenome *de novo* assembly, it is important to use alignment-based quality measures such as C1000, rather than more common N50 or N95, because of the ‘needle-in-a-haystack’ nature of metagenomic data. As we have demonstrated in Figures 2–4, these performance measures were generally consistent across datasets. These metrics can be efficiently calculated using MegaBLAST, which is used by Chimera.slayer (36).

When directly comparing the stool and nasal samples in the ‘*in silico*-virus spiked’ datasets, we find that the more complex background in the stool sample resulted in performance degradation in *de novo* assembly of the target BASV genome. Thus, rapid and efficient computational subtraction of human host reads (1) will likely remain a critical step in pathogen assembly from metagenomic data. Our analysis of the ‘*in silico*-virus spiked’ datasets also shows that an average sequence depth of at least 20× (10× in each direction for paired-end sequencing) is needed to recover 100% of a target genome from metagenomic NGS data. The actual minimum sequence depth required may be higher than 20× due to biases in the actual coverage achieved. Finally, although we have demonstrated the utility of the ensemble assembly strategy for the relatively small genomes corresponding to viruses, bacteria and the mitochondrial genome of the eukaryotic pathogen *N. fowleri*, this approach may not be suitable for much larger eukaryotic genomes, mainly because OLC assemblers such as Minimo and Cap3 will likely be unable to handle the increased computational load. As datasets become increasingly larger, the OLC assembly step may become the speed bottleneck, since the DBG assemblers and partitioning scheme are amenable to parallel computing with multi-core processors. The full results in tabular format were included in Supplementary Table S1. A diagram illustrating the calculation of performance metrics was included as Supplementary Figure S1.

## AVAILABILITY

The source code of our method can be obtained at http://ensembleassembly.sourceforge.net, or https://github.com/xutaodeng/EnsembleAssembler.

## SUPPLEMENTARY DATA

Supplementary Data are available at NAR Online.

SUPPLEMENTARY DATA
