# Supplementary material for: An ensemble strategy that significantly improves de novo assembly of microbial genomes from metagenomic next-generation sequencing data
Source: Nucleic Acids Res. 2015 Jan 13;43(7):e46. doi: 10.1093/nar/gkv002 (PMC4402509; doi:10.1093/nar/gkv002)
Supplement: SUPPLEMENTARY DATA [file supp_43_7_e46__index.html]

An ensemble strategy that significantly improves de novo assembly of microbial genomes from metagenomic next-generation sequencing data — SUPPLEMENTARY DATA 

# An ensemble strategy that significantly improves *de novo* assembly of microbial genomes from metagenomic next-generation sequencing data

## SUPPLEMENTARY DATA

**Files in this Data Supplement:**

- SUPPLEMENTARY DATA
- SUPPLEMENTARY DATA
