# Supplementary material for: An ensemble strategy that significantly improves de novo assembly of microbial genomes from metagenomic next-generation sequencing data
Source: Nucleic Acids Res. 2015 Jan 13;43(7):e46. doi: 10.1093/nar/gkv002 (PMC4402509; doi:10.1093/nar/gkv002)
Supplement: SUPPLEMENTARY DATA [file supp_gkv002_nar-02997-met-n-2014-File003.pptx]

## Slide 1
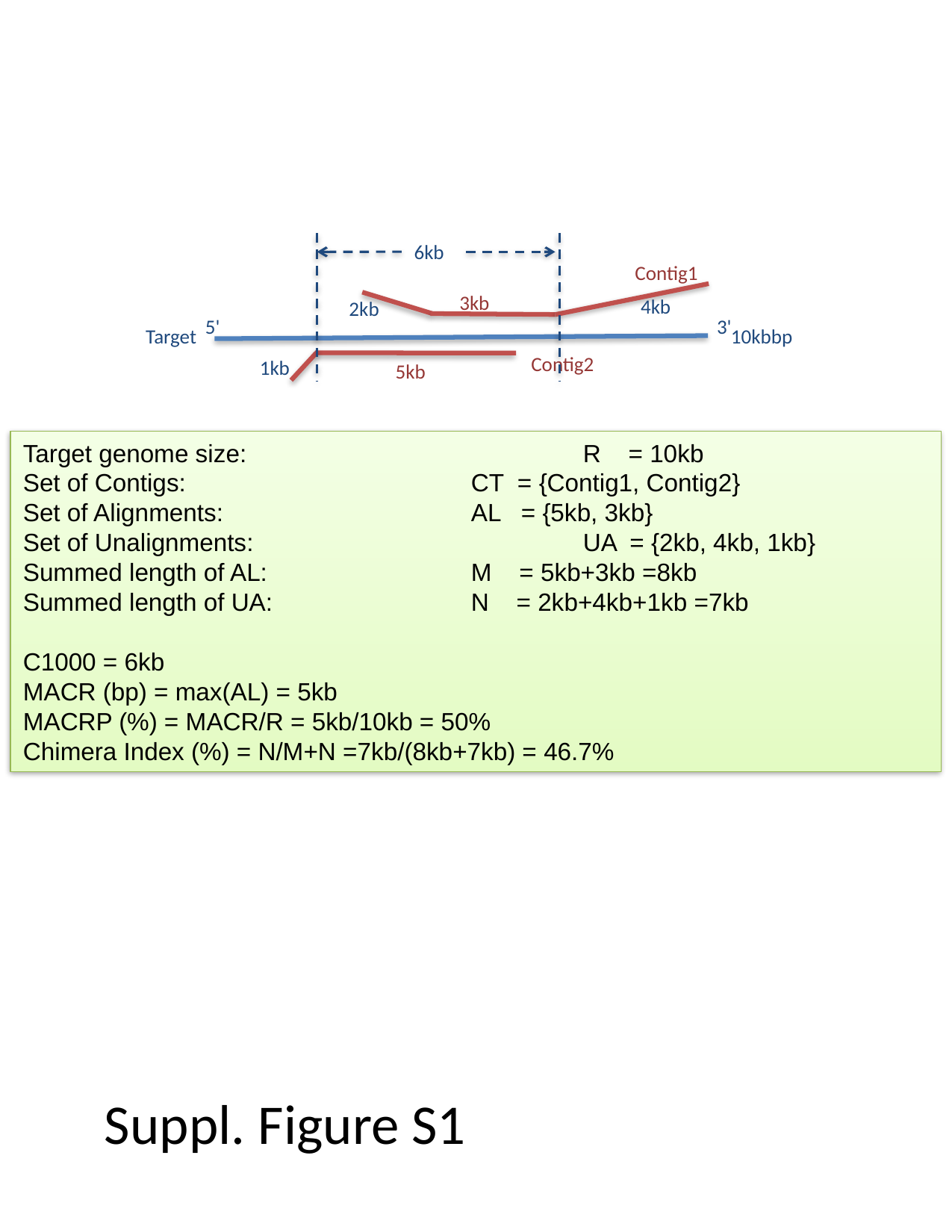

6kb
Contig1
3kb
4kb
2kb
3'
5'
Target
10kbbp
Contig2
1kb
5kb
Target genome size: 			R = 10kb
Set of Contigs: 			CT = {Contig1, Contig2}
Set of Alignments: 			AL = {5kb, 3kb}
Set of Unalignments: 			UA = {2kb, 4kb, 1kb}
Summed length of AL: 		M = 5kb+3kb =8kb
Summed length of UA: 		N = 2kb+4kb+1kb =7kb
C1000 = 6kb
MACR (bp) = max(AL) = 5kb
MACRP (%) = MACR/R = 5kb/10kb = 50%
Chimera Index (%) = N/M+N =7kb/(8kb+7kb) = 46.7%
Suppl. Figure S1
